# The development and validation of different decision-making tools to predict urine culture growth out of urine flow cytometry parameter

**DOI:** 10.1371/journal.pone.0193255

**Published:** 2018-02-23

**Authors:** Martin Müller, Ruth Seidenberg, Sabine K. Schuh, Aristomenis K. Exadaktylos, Clyde B. Schechter, Alexander B. Leichtle, Wolf E. Hautz

**Affiliations:** 1 Department of Emergency Medicine, Inselspital, Bern University Hospital, University of Bern, Bern, Switzerland; 2 Department of Anesthesiology, Inselspital, Bern University Hospital, University of Bern, Bern, Switzerland; 3 Department of Family & Social Medicine & Department of Epidemiology Population Health, Albert Einstein College of Medicine, Bronx, New York, United States of America; 4 Department of Clinical Chemistry, Inselspital, Bern University Hospital, University of Bern, Bern, Switzerland; Northwestern University, UNITED STATES

## Abstract

**Objective:**

Patients presenting with suspected urinary tract infection are common in every day emergency practice. Urine flow cytometry has replaced microscopic urine evaluation in many emergency departments, but interpretation of the results remains challenging. The aim of this study was to develop and validate tools that predict urine culture growth out of urine flow cytometry parameter.

**Methods:**

This retrospective study included all adult patients that presented in a large emergency department between January and July 2017 with a suspected urinary tract infection and had a urine flow cytometry as well as a urine culture obtained. The objective was to identify urine flow cytometry parameters that reliably predict urine culture growth and mixed flora growth. The data set was split into a training (70%) and a validation set (30%) and different decision-making approaches were developed and validated.

**Results:**

Relevant urine culture growth (respectively mixed flora growth) was found in 40.2% (7.2% respectively) of the 613 patients included. The number of leukocytes and bacteria in flow cytometry were highly associated with urine culture growth, but mixed flora growth could not be sufficiently predicted from the urine flow cytometry parameters. A decision tree, predictive value figures, a nomogram, and a cut-off table to predict urine culture growth from bacteria and leukocyte count were developed, validated and compared.

**Conclusions:**

Urine flow cytometry parameters are insufficient to predict mixed flora growth. However, the prediction of urine culture growth based on bacteria and leukocyte count is highly accurate and the developed tools should be used as part of the decision-making process of ordering a urine culture or starting an antibiotic therapy if a urogenital infection is suspected.

## Introduction

Urinary tract infections (UTI), ranging from uncomplicated cystitis to urosepsis, are amongst the most prevalent bacterial infections worldwide and are accountable for a large number of emergency consultations and hospitalizations [1, 2]. The direct and indirect costs for all urinary tract infections in the Unites States of America in 2010 were estimated to be about 2.3 billion dollars [3]. In Europe, one study estimated the total ambulatory costs of UTI in France to be about 58 Million Euro annually–nearly one Euro per inhabitant [4].

A patient with a suspicion of UTI will be treated with an empirical antibiotic therapy in accordance with international guidelines [5, 6]. As a result of the high incidence and this treatment recommendation, about 15% of all community-prescribed antibiotics are used for the treatment of UTI [7]. Considering the rising resistance rates, especially for *Escherichia coli* [8, 9]–by far the most common species found in UTI–a false positive diagnosis and subsequent overtreatment with antimicrobial treatment have to be minimized.

The gold standard for the diagnosis of a UTI is a positive urine culture [10]. In clinical practice this leads to a problem, as a urine culture takes several days to grow, but a decision about antimicrobial treatment often cannot be postponed. In the diagnosis of an uncomplicated UTI, the criteria on which the decision for antimicrobial treatment is based are mainly patient reported symptoms and urine dipsticks [10, 11]. Furthermore, microscopic examination of the urine sediment is possible. However, the frequently used urine dipstick suffers from a lack of sensitivity and specificity [12]; microscopic examinations are time consuming, expensive, and dependent on examiners’ experience [13]. In patients presenting with non-specific symptoms such as fever, nausea, abdominal tenderness or back pain, screening methods for the prediction of urine culture growth are essential to rule out/in urological infection. Thus, better decision aids are needed to predict probable future urine culture growth.

Automated urine analysis with urine flow cytometry was recently developed as a valid, inexpensive and quick screening prior to microscopic examinations [14–16]. Urine flow cytometry is fully automated and can count and classify the different urine particles such as epithelial cells, erythrocytes, cylinders, leukocytes, yeasts and bacteria with high correlation to manual microscopy [17]. The number of bacteria and leukocytes per μL is highly accurate and it has been shown to be predictive of future urine culture growth [18]. However, many different cut-offs exist, leading to confusion. Clinically applicable tools for decision-making have not yet reached their full potential.

Thus, the aim of this study was to develop different aids for decision-making to i) predict negative culture, ii) positive culture, and iii) mixed culture growth. Such instruments might have the potential to avoid antibiotic overtreatment on the one hand, and unnecessary ordering of urine culture on the other.

## Methods

### Study design and setting

The University Hospital of Bern (Inselspital) is one of the largest hospitals in Switzerland. More than 46,000 patients visit the facility each year, with a broad spectrum of diseases. This is a retrospective single center study to evaluate the use of prediction rules developed out of urine flow cytometry in decision-making for the diagnosis of UTI in the emergency department.

### Ethical considerations

The study was approved by the regional ethics committee of the Canton of Bern, Switzerland (KEK: 2016–01298).

### Data collection

A comprehensive medical report of every patient who presented at the emergency department is electronically stored. The urine of patients presenting with suspected UTI is routinely analyzed with urine flow cytometry. Furthermore, a urine culture is usually obtained. This procedure might differ if an uncomplicated cystitis is suspected and the diagnosis is based on symptoms or urine flow cytometry only.

Eligible (see below) patients were identified through a key-word search for “urine culture” with different semantic combinations in the health records, stored in the emergency department’s database (E-Care, ED 2.1.3.0, Turnhout, Belgium). The search was restricted to the period after the introduction of the urine flow cytometry to the time period starting on January 7^th^, 2016 and ending July 31^st^, 2016.

### Urine flow cytometry

The UX-2000 (Sysmex Corporation, Kobe, Japan) is a fully automated urine analysis that quantifies different urine parameter via fluorescence flow cytometry such as: erythrocytes, leukocytes, epithelial cells, casts, bacteria, mucus, sperms, crystals, round epithelial cells, cylinders, and pathological cylinders.

At least 4 mL of urine is needed for analysis. The analysis takes four minutes and the results are available to the physician within 30 minutes, just after validation by the lab technician. The automated counts of the UX-2000 have shown a good correlation to manual microscopic counts [17]. All flow analyses were performed in an ISO 17025 accredited laboratory (Center of Laboratory Medicine, Inselspital).

### Urine culture

Nurses and laboratory staff are regularly trained to ensure high quality standards to obtain 5mL of clean midstream/catheter urine in a vacutainer urine collection tube with boric acid (urine culture kit) and to send it to the laboratory within two hours.

In daily practice the urine culture is prepared directly until 4 pm with 5μL for CHROMagar and CNA-agar (colistin and nalidixic acid-agar) and incubated at 35°C without CO_2._ Antimicrobial bacterial activity is proven by *Bacillus subtilis*. Identification of the microorganism is realized with MALDI-TOF, resistance examination with the Kirby Bauer method. After 24 hours and also after 48 hours the results are taken and read off.

### Eligibility criteria

All adult patients found through the key-word search were included when they had a urine culture and a urine flow cytometry obtained in the first 24h of their visit to the emergency department. Patients younger than 16 years old and those without a urine flow cytometry and/or without a urine culture were excluded.

### Study outcomes

The study outcome was urine culture growth. According to the European association of Urology, there is a large range of a colony forming unit (cfu) cut-offs defining a positive urine culture ranging from 10^2^ cfu/mL in catheter urine samples of symptomatic patients to 10^5^ cfu/mL in a spontaneously voided urine sample in asymptomatic patients [6].

Significant urine culture growth is defined here as at least 10^4^ cfu/mL, because this limit i) represents the cut-off for significant bacterial growth in all complicated UTI, even in straight urine catheter samples [6], ii) is used in most of the urine flow cytometry studies [18], and iii) is often the minimum bacterial growth that is generally reported by clinical microbiology laboratories. A mixed culture was defined as a significant bacterial growth (≥10^4^ cfu/mL) with a mixed growth pattern.

Three outcome variables were defined. A categorical variable with the levels “no significant culture growth”, “significant mixed flora growth” and “significant culture growth” was defined. Furthermore, two binary variables were created that classify the urine sample into i) “positive culture growth” (independently of mixed flora growth) vs. “no growth”and ii) “mixed flora growth” vs. “no mixed flora growth”.

### Data extraction

The following data for eligible patients were anonymized and extracted from the medical record of the emergency department into Microsoft Excel for Mac 2011 (Microsoft Corporation, USA): patient demographics such as age and sex, patient-reported data such as the presence of dysuria and urinary frequency, clinical findings such as suprapubic/flank/abdominal pain and fever, patient comorbidities, the discharge diagnosis group [19] as well as the urogenital diagnosis, if any, at discharge.

Urine flow cytometry results were automatically extracted and the number and species of an obtained urine culture were manually extracted from the laboratory database (Xserv.4, ixmid Software Technologie GmbH, Germany).

### Statistical analysis

Statistical analysis was mainly performed using Stata 13.1 (StataCorp, College Station, Texas, USA). The whole sample was randomly divided into two group-sets: a training set and a validation set with a ratio of 70:30. Continuous variables (e.g. age) were presented with mean and standard deviation (SD) while categorical data were described as the absolute number and percent.

The association of mixed culture growth as well as positive culture growth with urine flow cytometry parameters as predictors were tested using logistic regression.

Different statistical approaches to predict a positive urine culture from the urine flow cytometry parameters bacteria and leukocytes were developed using the training set and validated with the validation set:

- A colored scatter plot was generated out of the urine flow cytometry parameter to predict a positive urine culture (bacteria and leukocytes).
- A decision tree was development and its validation presented using SPSS (IBM Corp. Released 2016. IBM SPSS Statistics for Windows, Version 24.0. Armonk, NY: IBM Corp) with a Chi-square Automatic Interaction Detectors (CHAID) algorithm.
- A nomogram was created (training set) from a bootstrapped logistic regression and its predictive values for different predicted probabilities are presented (validation set).
- Predictive values of the validation set for different bacteria and leukocytes cut-offs for a positive urine culture test found by analysis of the training set (or published previously by other studies) are summarized.

Predictive values were presented with the associated 95% confidence interval (CI). A P-value of less than 0.05 was defined as statistically significant and P < 0.001 as highly significant.

The initial idea of predicting the categorical culture growth as 1) “no significant culture growth”, 2) “significant, mixed-culture growth”, 3) “significant, non-mixed culture growth” out of the urine flow cytometry parameter was discarded because the outcome mixed culture growth could not be adequately predicted (see below).

## Results

### Patient characteristics

**Fig 1:**
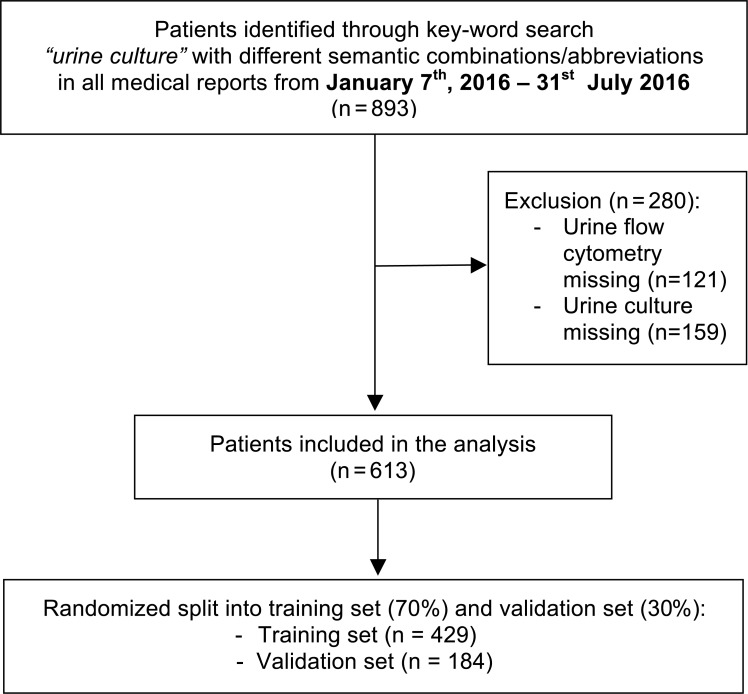
Flowchart of the study.

Six hundred and thirteen (n = 613) patients fulfilled the eligibility criteria and were included in the analysis. The sample was randomly divided into a ratio of 70:30 into a training set (n = 429) and a validation set (n = 184). The flowchart of the selection process is shown in Fig 1.

The mean age was 59.5 (SD 19.6) years and 48.5% of the patients were female. Clinical and patient-reported data that were often found were fever (28.3%), abdominal or flank pain (cumulative 33.2%), and dysuria (18.1%). A urogenital diagnosis at discharge was identified in 70.2% of the cases. Possible urogenital infection/urosepsis (29.5%) was the most frequently documented urogenital diagnosis. A detailed summary of the patients’ characteristics is shown in Table 1.

**Table 1:** Patient characteristics.

		Training set (n = 429)		Validation set (n = 184)		Total (n = 613)	
**Demographic data**							
	Age, mean (SD)	59.6	(19.2)	59.4	(20.9)	59.5	(19.6)
	Sex, female, n (%)	212	(49.4)	85	(46.2)	297	(48.5)
**Anamnestic / clinical data, n (%)**							
	Dysuria	71	(16.6)	41	(22.3)	112	(18.1)
	Urinary frequency	56	(13.1)	22	(12.0)	78	(12.7)
	Abdominal pain	84	(19.6)	50	(27.2)	134	(21.9)
	Flank pain	50	(11.7)	19	(10.3)	69	(11.3)
	Fever (>38.2°C)	107	(30.0)	36	(24.2)	143	(28.3)
	Suprapubic pain	45	(10.6)	20	(11.0)	65	(10.7)
**Comorbidity, n (%)**							
	Diabetes mellitus Typ1/2	88	(20.6)	48	(26.2)	136	(22.3)
	Structural urogenital disease^a^	111	(26.0)	42	(23.0)	153	(25.0)
	Bladder catheter	59	(13.8)	25	(13.7)	84	(13.8)
	Immunosuppression	151	(35.3)	64	(35.0)	215	(35.2)
	Prior antibiotic therapy	108	(25.2)	49	(26.6)	157	(25.6)
**Urogenital diagnosis, n (%)**							
	Asymptomatic bacteriuria	4	(0.9)	3	(1.6)	7	(1.1)
	Uncomplicated UTI	59	(13.8)	23	(12.5)	82	(13.4)
	Complicated UTI	25	(5.8)	20	(10.9)	45	(7.3)
	Pyelonephritis	33	(7.7)	11	(6.0)	44	(7.2)
	Possible urog. infection/ sepsis	128	(29.8)	53	(28.8)	181	(29.5)
	Urosepsis	47	(11.0)	12	(6.5)	59	(9.6)
	Urethritis/Balanitis	2	(0.5)	1	(0.5)	3	(0.5)
	Urinary retention	4	(0.9)	1	(0.5)	5	(0.8)
	Prostatitis	5	(1.2)	7	(3.8)	12	(2.0)
	Epididymitis/orchitis	5	(1.2)	0	(0.0)	5	(0.8)
	Urolithiasis	4	(0.9)	3	(1.6)	7	(1.1)
	Glomerulonephritis	2	(0.5)	1	(0.5)	3	(0.5)
	Other urogenital diagnosis	2	(0.5)	1	(0.5)	3	(0.5)
	No specific urog. diagnosis	109	(25.4)	48	(26.1)	157	(25.6)
	Infectious disease	26	(23.9)	15	(31.3)	41	(26.1)
	Respiratory problem	25	(23.0)	8	(16.7)	33	(21.0)
	Gastrointestinal problem	15	(13.8)	10	(20.8)	25	(15.9)
	Neurological problem	16	(14.7)	5	(10.4)	21	(13.4)
	Other	27	(24.6)	10	(20.8)	37	(23.6)
**CFU/mL in urine culture, n (%)**							
	0	127	(29.6)	65	(35.3)	192	(31.3)
	100	1	(0.23)	0	(0.0)	1	(0.2)
	1000	123	(28.7)	50	(27.2)	173	(28.2)
	10000	89	(20.8)	32	(17.4)	121	(19.7)
	100000	89	(20.8)	37	(20.1)	126	(20.6)
**Administrative data**							
	Hospitalization	324	(75.5)	127	(69.0)	451	(73.6)

**Abbreviations:** CFU, central-forming unit; UTI, urinary tract infection.

^a^most often past prostate operations.

Two hundred and forty-seven (40.6%) urine cultures met the criteria for a positive culture with at least 10^4^ cfu/mL. *Escherichia coli* was found in 48.6% of the positive cultures, followed by *Klebsiella pneumoniae* (5.7%), and *Staphylococcus aureus* (4.5%). A mixed culture was found in 17.8% (see Table 2).

**Table 2:** Distribution of species of positive culture (≥10^4^), n = 247 (40.2%).

Species	Training set		Validation set		Total	
*Escherichia coli*	86	(48.3)	34	(49.3)	120	(48.6)
*Klebsiella pneumoniae*	9	(5.0)	5	(7.3)	14	(5.7)
*Staphylococcus aureus*	8	(4.5)	3	(4.4)	11	(4.5)
*Enterococcus faecalis*	6	(3.4)	2	(2.9)	8	(3.2)
*Pseudomonas aeruginosa*	6	(3.4)	1	(1.5)	7	(2.8)
*Coagulase-negative Staphylococci*	1	(0.6)	4	(5.8)	5	(2.0)
*Lactobacillus species*							4	(2.3)	0	(0)	4	(1.6)
*Enterobacter cloacae*	3	(1.7)	1	(1.5)	4	(1.6)
*Klebsiella oxytoca*	4	(2.3)	0	(0.0)	4	(1.6)
*Aerococcus urinae*	2	(1.1)	1	(1.5)	3	(1.2)
Mixed Flora	34	(19.1)	10	(14.5)	44	(17.8)
Other	15	(8.3)	8	(11.3)	23	(8.4)
**Total**	178	(100.0)	69	(100.0)	247	(100.0)
p-value: 0.448							

A majority of the patients (73.6%) were hospitalized.

### Urine culture growth

The number of leukocytes and bacteria in urine flow cytometry showed a highly significant association (p<0.0001) in logistic regression with a positive urine culture (independently of the species or mixed flora) and all urine flow cytometry parameters as independent parameters. Cylinders (p = 0.016), yeasts (p = 0.027), and pathological cylinders (p = 0.006) in the urine flow cytometry presented significant associations with positive urine culture, but the association did not remain significant when restricting the analysis to positive urine culture without mixed flora and adding an interaction term between bacteria and leukocytes; hence they were not included further in the decision-making aids. The area under the receiver-operating curve (ROC) in the whole set to predict culture growth out of bacteria and leukocyte count was 0.93 (95% CI: 0.90, 0.94).

**Fig 2:**
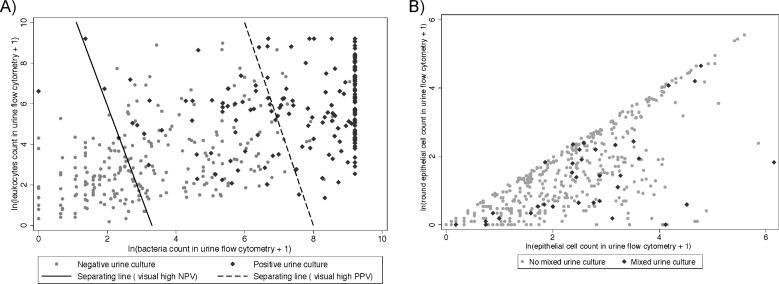
Scatter plots for urine flow cytometry parameter of the training set, n = 429. **Positive (A) vs. mixed (B) urine culture (≥10**^**4**^**) are colored in black. In A: left of the solid line most of the observations showed no growth. Setting a test cut-off for bacteria and leukocytes in urine flow cytometry left of the line will lead to a high negative predictive value (NPV) for urine culture growth; vice versa, cut-off values defined by the dotted line will lead to a high positive predictive value (PPV).** For a better graphical representation the number of bacteria and leukocytes, respectively round epithelial cells and epithelial cells (per μL) were *ln*-transformed.

**Fig 3:**
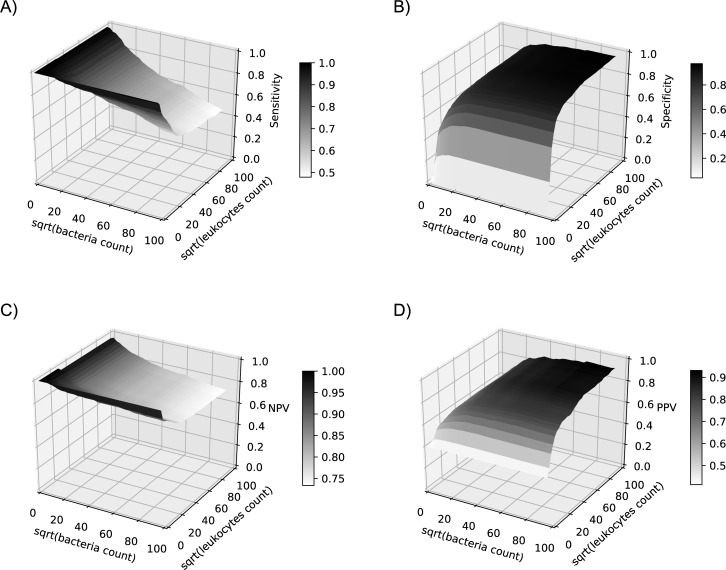
A) Sensitivity, B) Specificity, C) negative predictive value (NPV) and D) positive predictive value (PPV) for a positive urine culture for different cut-offs of bacteria and leukocytes (square root-transformed) of the whole data set, n = 613.

Fig 2A plots the numbers of bacteria and leukocytes in cultures with and without growth. Parameter combinations of leukocyte and bacteria counts left of the solid line generally have no culture growth whereas parameter combinations right of the dotted line have. A decision test that is positive if the bacteria and leukocytes count of an urine flow cytometry are right of the dotted line–which is equivalent to the following equation: *ln(lecucytes+1) > 40–5 x ln(bacteria+1)–*therefore has a high positive predictive value for positive culture growth (see Table 3). The relationship between predictive values and different cut-offs of bacteria and leukocyte counts for a positive decision test is shown in Fig 3.

**Table 3:** Predictive values for different cut-offs (growth probability: Low, medium, high) for urine flow cytometry for the number of bacteria/μL and leukocytes/μL predicting a positive urine culture (≥10^4^), validation set n = 184.

** **	Test positive	Remark	Growth prob.	Predictive values	
	Bacteria > 23.7	Maximal Bacteria with a sensitivity > 95%.	low	SE:	98.6 (92.2, 100,0)
				SP:	56.5 (47.0, 65.7)
				PPV:	57.6 (48.2, 66.7)
				NPV:	98.5 (91.8, 100)
	ln(Leukocytes+1) > 15–4.54 x ln(Bacteria+1)	Point right of the solid line (Fig 2)	low	SE:	100.0 (94.8, 100.0)
				SP:	49.6 (40.1, 59.0)
				PPV:	54.3 (45.3, 63.2)
				NPV:	100.0 (93.7, 100)
	Bacteria>90 OR Leukocytes>70	Sensitivity>99% & highest specificity	low	SE:	94.2 (85.8, 98.4)
				SP:	64.3 (54.9, 73.1)
				PPV:	61.3 (51.4, 70.6)
				NPV:	94.9 (87.4, 98.6)
^a^	Bacteria>125 OR Leukocytes>17	In-house reference	low	SE:	98.0 (95.3, 99.3)
				SP:	48.9 (43.7, 54.2)
				PPV:	56.4 (51.6, 61.2)
				NPV:	97.3 (93.8, 99.1)
^a^	Bacteria>125 OR Leukocytes>40	Manoni, Fornasiero [16]	low	SE:	97.2 (94.2, 98.9)
				SP:	56.6 (51.3, 61.7)
				PPV:	60.2 (55.2, 65.0)
				NPV:	96.7 (93.4, 98.7)
^a^	Bacteria>170 OR Leukocytes>150	De Rosa, Grosso [15]	low	SE:	93.9 (90.2, 96.6)
				SP:	69.3 (64.4, 74.1)
				PPV:	67.4 (62.2, 72.4)
				NPV:	94.4 (91.0, 96.8)
^a^	Bacteria>405 OR Leukocytes>16	Jolkkonen, Paattiniemi [31]	low	SE:	96.0 (92.7, 98.0)
				SP:	50.5 (45.3, 55.8)
				PPV:	56.7 (51.8, 61.5)
				NPV:	94.9 (90.8, 97.5)
	Bacteria >724.3	Bacteria with the highest Youden-Index	med	SE:	73.9 (61.9, 89.7)
				SP:	91.3 (84.6, 95.8)
				PPV:	83.6 (71.9, 91.8)
				NPV:	85.4 (77.9, 91.1)
	Bacteria >900 OR Leukocytes>270	Combination of bacteria & leukocytes with the highest Youden-Index	med	SE:	85.5 (75.0, 92.8)
				SP:	82.6 (74.4, 89.0)
				PPV:	74.7 (63.6, 83.8)
				NPV:	90.5 (83.2, 95.3)
	Bacteria > 2534	Minimal Bacteria with a specificity > 95%	high	SE:	62.3 (49.8, 73.7)
				SP:	94.8 (89.0, 98.1)
				PPV:	87.8 (75.2, 95.4)
				NPV:	80.7 (73.1, 87.0)
	Bacteria>890 OR Leukocytes>2330	Specificity>90% & lowest bacteria count in the training set	high	SE:	94.2 (85.8, 98.4)
				SP:	64.3 (54.9, 73.1)
				PPV:	61.3 (51.4, 70.6)
				NPV:	94.9 (87.4, 98.6)
	ln(Leukocytes+1) > 40–5 x ln(Bacteria+1)	Point right of the dotted line (Fig 2)	high	SE:	73.9 (61.9, 83.7)
				SP:	93.0 (86.8, 96.6)
				PPV:	86.4 (75.0, 94.0)
				NPV:	85.6 (78.2, 91.2)

**Abbreviations:** ln, logarithmus naturalis; NPV/PPV, negative/positive predictive value; SE, sensitivity, SP, specificity; prob., probability.

^a^ external cut-off values; validated on the whole sample.

### Mixed flora

Epithelial cells (p = 0.012), round epithelial cells (p = 0.012), and cylinder (p = 0.006) were associated with mixed flora growth. The area under the receiver operating characteristic curve (AUC) in the whole set to predict mixed flora growth out of the identified predictors was 0.66 (95% 0.61, 0.70).

The first attempt was to model the categorical outcome levels i) no growth, ii) mixed flora growth, and iii) positive culture growth out of the identified five urine flow cytometry parameters. The results of these models did not usefully predict mixed flora growth. Fig 2B illustrates the missing predictive value of epithelial cells to predict mixed culture growth. The illustration is similar in a three-dimensional plot additionally incorporating cylinders (see https://figshare.com/articles/Figure_pdf/5873799).

The decision-making tools presented below were therefore restricted to predicting positive urine culture growth (independently of mixed flora growth) vs. no growth out of the bacteria and leukocyte counts.

### Decision tree

**Fig 4:**
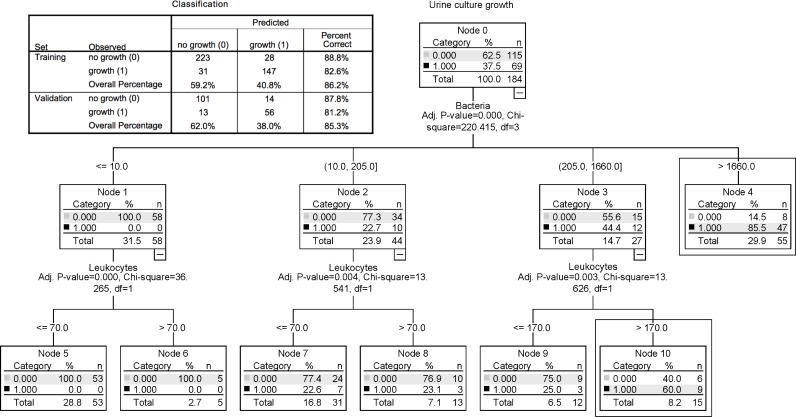
Validation of the developed CHAID-classification tree with the validation set, n = 184, and a comparison of the classification of the training and validation data set. **The framed nodes predict urine culture growth.** The units of bacteria and leukocytes are per μL.

A decision tree using the Chi-square automatic interaction detection (*CHAID*) was build using the training set. Culture growth vs. no. growth (binary coded) was the dependent variable and the counts of leukocytes and bacteria were the independent variables. The validation of the tree in the validation set is shown in Fig 4. The percent of the cultures that are correctly classified if the culture has shown bacterial growth was 81.2% (node 4 and 10) while no growth culture were classified correctly over all other nodes in 87.8% of the observations leading to an overall correct prediction of 85.3%.

### Nomogram

The training set was used to create a nomogram from a bootstrapped logistic regression predicting positive urine culture from bacteria and leukocyte count. The area under the receiver-operating characteristic curve for these predictions was 0.92 (95% CI: 0.89, 0.95) in the training set and 0.93 (95% CI: 0.89, 0.96) in the validation set.

**Fig 5:**
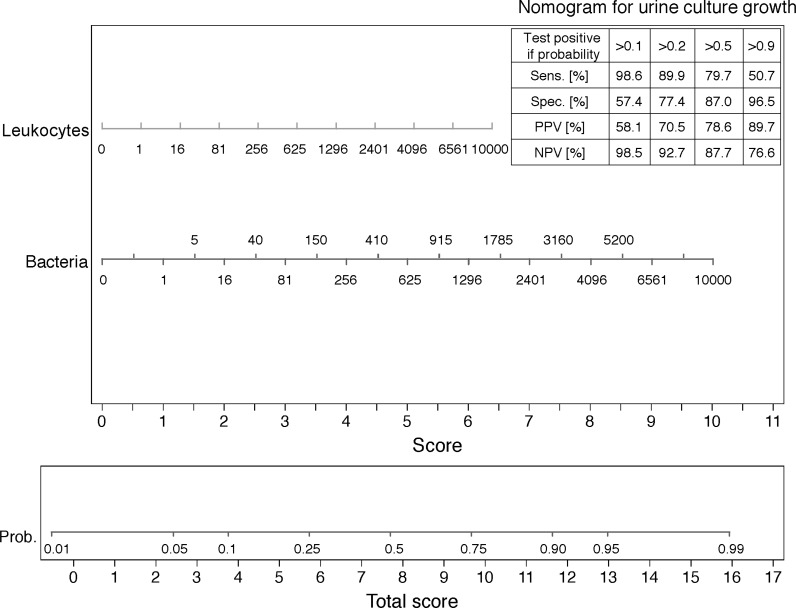
Nomogram for urine culture growth based on the training sample (n = 429) and predictive values for different predicted possibility cut-offs for a positive test (test pos.) based on the validation sample (n = 184). **N** Example: Considering the urine flow cytometry of a patient shows 80 leukocytes/μL and bacteria16/μL. Eighty leukocytes/μL correspond to ≈2.0 points on the score axis, 16 bacteria/μL correspond to ≈2.0 point. Thus, the total score, the sum of the single scores is 4.0 (2.0+2.0). The predicted probability of urine culture growth can be read off the probability axis. Four points on the probability axis correspond to a urine culture growth of about 10%. A test that is defined positive, when the predicted probability of culture growth is greater than 10% (table right corner), has a sensitivity of 98.6%. Thus, urine culture growth is very unlikely and ordering a urine culture not recommended. Remark: The axis of leukocytes and bacteria per μL are (.)^0.25^- transformed to obtain predictive probabilities between 0.01 and 0.99.

Fig 5 shows the obtained nomogram and predictive values for different probability cut-offs (validation set). For both, a given number of bacteria and leukocytes, a related score is assigned. Out of the sum of the scores, the total score is obtained. From the probability axis the probability for culture growth for the obtained total score can be read off. Each given probability of urine culture growth leads to different predictive values. For example, a test that is defined as positive if the predicted probability of urine culture growth is higher than 10% has a sensitivity of 98.6% (95% CI: 92.2%-100%) and a specificity of 57.4% (95%: 47.8%, 66.6%). For a sample calculation example see Fig 5.

### Cut-off table

As illustrated in Fig 3, many different combinations of leukocytes and bacteria count cut-offs often have similar predictive values. Table 3 shows different cut-off values developed out of the training set or suggested in the literature and the corresponding predictive values validated with the validation set, or the whole sample in case of external suggested parameters.

## Discussion

### Statement of principal findings

A retrospective analysis of patients presenting to an emergency department was performed to predict urine culture growth from urine flow cytometry parameters and different decision-making tools were developed and validated. While the number of leukocytes and bacteria were strongly associated with positive culture growth, mixed flora growth could not be sufficiently predicted from the urine flow cytometry parameters. To our knowledge, this is the first study that developed and validated different decision-making tools i.e. a decision tree, predictive value figures, a nomogram, and a cut-off table to predict urine culture growth out of bacteria and leukocyte count of urine flow cytometry.

### Results in context

Polymicrobial bacteriuria or mixed flora is usually considered as contamination even though in special situations such as long-term catheterization it can be of significance [20]. In this trial, we tried to predict mixed flora growth out of epithelial and round epithelial cells. One reason for the failure to predict mixed flora might be the fact that squamous epithelial cells, which were traditionally thought to have a higher predictive value than epithelial cells for instance [21], cannot be determined by UX-2000. The predictive performance of squamous cells with future generations of urine flow cytometer such as UF-4000 (Sysmex, Kobe, Japan), which allows the quantification of squamous cells, needs to be further studied. However, even squamous cells identified through microscopy yield a poor performance in predicting mixed flora [22]. Thus, a different approach might be required. Two trials used different patterns of bacteria fluorescent light to predict bacterial morphologies and mixed flora correctly using UF1000i (Sysmex, Kobe, Japan) [23, 24]. While Yang, Yang [24] concluded that results of laser flow cytometry predict growth of mixed flora, the results of Geerts, Jansz [23] showed underperformance in the category mixed flora. Further studies are needed to clarify the results of this promising approach and give recommendations for the use in clinical practice which is of special use in the emergency setting where the contamination rate is often found to be high [25].

Several studies focused on finding the “optimal” cut-off for different parameters that can be found by flow cytometry to predict urine culture growth [18]. Most of them include count of bacteria, leukocytes or the combination of both to predict urine culture growth; especially high sensitivity cut-off values to rule out future urine culture growth were presented using different cytometers such as UX-2000, UF-1000i, UF-100, Accuri C6 and others [15, 26–30]. With a cut-off value of 170 bacteria/μL and 150 leukocytes/μL, a sensitivity of 98.8%, a specificity of 76.5%, a negative predictive value of of 99.5% and four false negative results could be obtained (1.2%), avoiding the culture in 57.1% of samples [15]. The comparison with high-sensitivity cut-offs found in other studies is tricky as they often used different cut-off criteria for bacterial growth (e.g. 10^5^ cfu/mL [16] or more complex criteria [31]), other study populations (e.g. including outpatient and general practitioner patients [30]), and other types of urine flow cytometer [15]. However, high sensitivity could be shown with our parameter too, even with a cut-off of 10^4^ cfu/mL [15, 16, 31]. Recently, a Swedish study presented a linear discriminant analysis using bacteria and leukocytes on a log scale [29] similar to Fig 2A. The parameters were slightly different from the parameters presented in this article, which might be due to another cut-off of urine culture bacterial growth (≥10^3^ cfu/mL) and the use of Sysmex UF1000i. While such an approach is powerful by covering many different bacteria/leukocyte-combinations, the equation might not be useful in clinical practice due to its complexity.

Shang, Wang [18] concluded, in their systematic review on cut-off values for bacteria and leukocytes to predict urine culture growth focusing on UF-100 and UF-1000i, that the study populations were often not representative of UTI patients. This is a major limitation of their review as the disease prevalence and the characteristics of the population have to be taken into account, when interpreting the results [16]. In our study, the population consisted of patients presenting at the emergency department of a university hospital with a suspected UTI–a population that is heterogeneous, and also includes polymorbid, transplanted as well as immunosuppressed patients. One trial studied febrile patients in an emergency department. The authors presented a larger high-sensitivity bacteria cut-off compared to other trials to rule out UTI in febrile patients [32]. Further research on special subgroups of patients is required to improve the decision-making in specific scenarios.

Different tools were created and validated including a comprehensive nomogram that is detached from the “optimal” cut-off illusion and may be used for the interpretation of the results of the UX-2000 to evaluate a patient at the emergency department with a suspected urogenital infection. These tools are an aid for decision-making, when flow cytometry is used as one piece of the puzzle to lead to a diagnosis, treatment, or to decide if further diagnostic investigation is necessary. The decision about which tool to use is of individual preference.

### Strengths and weaknesses of the study

This study is a retrospective study of laboratory data and health records. Information bias of the independent variable (urine flow cytometry parameter) and outcome variables (urine culture growth) is unlikely due to the use of laboratory tests that are regularly validated. Thus, high data quality in these variables can be assumed. However, clinical data that are used to describe the study sample are based on health records and completeness cannot be assured. Furthermore, selection bias might be a limitation of this trial, especially because more than 75% of the patients included in this study were subsequently hospitalized. Whether and how our results generalize to a healthier population remains to be investigated. The decision to order a urine culture was in the responsibility of the physician in charge. Thus, inter-individual variations might have led to selection bias.

An interesting question concerns the predictive value of urine flow bacteria and leucocytes for urine culture growth in specific subgroups of patients e.g. in which the clinical suspicion of a urosepsis was high. However, we are not able to analyze our data in that regard because the discharge diagnosis were made by the physician, thus the urine flow cytometry was taken into account in that diagnosis and all included patients were initially under the suspicion of having a UTI.

A broad search algorithm was used to identify all patients with an obtained urine culture to ensure a small number of missing eligible patients.

Despite frequent training of the nurses to educate a patient in the procedure of giving a clean urine sample, the quality of urine culture reflects the quality of taking urine cultures in an emergency department with an increased rate of mixed flora culture. External validity can only be assured with respect to a definition of a positive urine culture of at least 10^4^ cfu/mL, the urine flow cytometer UX-2000 and to patient populations with a high number of complicated UTI and hospitalization rate.

### Implications for clinicians

Medical decision-making aids such as scores, flow-charts, and algorithms are nowadays an essential element in daily routine and are thought to increase the quality of care and support evidence-based treatment [33]. This article provides the physician with different designed tools in tabularized form, in the form of a decision tree, as well as a graphical calculating device (nomogram) for use in clinical practice. Cut-off values with high sensitivity and negative predictive values were presented. Thus, the tools have the potential to reduce unnecessary prescription of antibiotics and to avoid initiating unnecessary urine cultures.

### Unanswered questions and future research

Although studies have shown an economic benefit of the use of urine flow cytometry before urine culture [34], the impact on the prescription of antibiotics remains unknown.

Furthermore, there is a lack of studies that focus on urine flow cytometry cut-offs in specific clinical subgroups e.g. febrile [32] and especially immunosuppressed patients. In the setting of immunosuppressed patients predicting mixed flora growth is particular important. Thus, future research is needed to evaluate the predictive performance of new generation cytometer especially of squamous cells, which are quantified e.g. in UF-4000, or use other approaches to predict mixed flora culture.

## Conclusions

Urine flow cytometry parameters fail to predict mixed flora growth. However, the prediction of urine culture growth from bacteria and leukocytes is highly accurate and several tools were presented that can be used in the decision process of initiating an urine culture or starting an antibiotic therapy for suspected urogenital infection.

## Supporting information

S1 File Dataset of the study. (XLS)

## References

[1] Cardwell SM, Crandon JL, Nicolau DP, McClure MH, Nailor MD. Epidemiology and economics of adult patients hospitalized with urinary tract infections. Hosp Pract (1995). 2016;44(1):33–40. doi: 10.1080/21548331.2016.1133214 . 26673518 10.1080/21548331.2016.1133214

[2] Schappert SM, Rechtsteiner EA. Ambulatory medical care utilization estimates for 2007. Vital Health Stat 13 2011;(169):1–38. . 21614897

[3] Foxman B. Urinary tract infection syndromes: occurrence, recurrence, bacteriology, risk factors, and disease burden. Infect Dis Clin North Am. 2014;28(1):1–13. doi: 10.1016/j.idc.2013.09.003 . 24484571 10.1016/j.idc.2013.09.003

[4] Francois M, Hanslik T, Dervaux B, Le Strat Y, Souty C, Vaux S, et al The economic burden of urinary tract infections in women visiting general practices in France: a cross-sectional survey. BMC Health Serv Res. 2016;16(a):365 doi: 10.1186/s12913-016-1620-2 ; PubMed Central PMCID: PMC4977873. 27507292 10.1186/s12913-016-1620-2 PMC4977873

[5] Gupta K, Hooton TM, Naber KG, Wullt B, Colgan R, Miller LG, et al International clinical practice guidelines for the treatment of acute uncomplicated cystitis and pyelonephritis in women: A 2010 update by the Infectious Diseases Society of America and the European Society for Microbiology and Infectious Diseases. Clin Infect Dis. 2011;52(5):e103–20. doi: 10.1093/cid/ciq257 . 21292654 10.1093/cid/ciq257

[6] Grabe M, Bartoletti R, Bjerklund Johansen TE, Cai T, Çek M, Köves B, et al Guidelines on urological infections. 2015 [cited 24.09.2017]. Available from: https://uroweb.org/wp-content/uploads/19-Urological-infections_LR2.pdf.

[7] Mazzulli T. Antimicrobial resistance trends in common urinary pathogens. Can J Urol. 2001;8 Suppl 1:2–5. . 11442990

[8] Bader MS, Loeb M, Brooks AA. An update on the management of urinary tract infections in the era of antimicrobial resistance. Postgrad Med. 2017;129(2):242–58. doi: 10.1080/00325481.2017.1246055 . 27712137 10.1080/00325481.2017.1246055

[9] European Centre for Disease Prevention and Control. Summary of the latest data on antibiotic resistance in the European Union Stockholm: ECDC; 2016.

[10] Schmiemann G, Kniehl E, Gebhardt K, Matejczyk MM, Hummers-Pradier E. The diagnosis of urinary tract infection: a systematic review. Dtsch Arztebl Int. 2010;107(21):361–7. doi: 10.3238/arztebl.2010.0361 ; PubMed Central PMCID: PMC2883276. 20539810 10.3238/arztebl.2010.0361 PMC2883276

[11] Poon E, Self L, McLeod SL, Caine S, Borgundvaag B. Uncomplicated urinary tract infections in the emergency department: a review of local practice patterns. CJEM. 2017:1–6. doi: 10.1017/cem.2017.39 . 28587696 10.1017/cem.2017.39

[12] Mambatta AK, Jayarajan J, Rashme VL, Harini S, Menon S, Kuppusamy J. Reliability of dipstick assay in predicting urinary tract infection. J Family Med Prim Care. 2015;4(2):265–8. Epub 2015/05/08. doi: 10.4103/2249-4863.154672 PubMed Central PMCID: PMC4408713. 25949979 10.4103/2249-4863.154672 PMC4408713

[13] Shayanfar N, Tobler U, von Eckardstein A, Bestmann L. Automated urinalysis: first experiences and a comparison between the Iris iQ200 urine microscopy system, the Sysmex UF-100 flow cytometer and manual microscopic particle counting. Clin Chem Lab Med. 2007;45(9):1251–6. Epub 2007/07/20. doi: 10.1515/CCLM.2007.503 17635081 10.1515/CCLM.2007.503

[14] Boonen KJ, Koldewijn EL, Arents NL, Raaymakers PA, Scharnhorst V. Urine flow cytometry as a primary screening method to exclude urinary tract infections. World J Urol. 2013;31(3):547–51. Epub 2012/05/17. doi: 10.1007/s00345-012-0883-4 22588552 10.1007/s00345-012-0883-4

[15] De Rosa R, Grosso S, Bruschetta G, Avolio M, Stano P, Modolo ML, et al Evaluation of the Sysmex UF1000i flow cytometer for ruling out bacterial urinary tract infection. Clin Chim Acta. 2010;411(15–16):1137–42. Epub 2010/04/03. doi: 10.1016/j.cca.2010.03.027 20359474 10.1016/j.cca.2010.03.027

[16] Manoni F, Fornasiero L, Ercolin M, Tinello A, Ferrian M, Hoffer P, et al Cutoff values for bacteria and leukocytes for urine flow cytometer Sysmex UF-1000i in urinary tract infections. Diagn Microbiol Infect Dis. 2009;65(2):103–7. doi: 10.1016/j.diagmicrobio.2009.06.003 . 19748419 10.1016/j.diagmicrobio.2009.06.003

[17] Wesarachkitti B, Khejonnit V, Pratumvinit B, Reesukumal K, Meepanya S, Pattanavin C, et al Performance Evaluation and Comparison of the Fully Automated Urinalysis Analyzers UX-2000 and Cobas 6500. Lab Med. 2016;47(2):124–33. doi: 10.1093/labmed/lmw002 . 27069030 10.1093/labmed/lmw002

[18] Shang YJ, Wang QQ, Zhang JR, Xu YL, Zhang WW, Chen Y, et al Systematic review and meta-analysis of flow cytometry in urinary tract infection screening. Clin Chim Acta. 2013;424:90–5. Epub 2013/06/01. doi: 10.1016/j.cca.2013.05.014 23721948 10.1016/j.cca.2013.05.014

[19] Müller M, Klingberg K, Srivastava D, Exadaktylos AK. Consultations by asylum seekers: recent trends in the emergency department of a Swiss university hospital. PLoS one. 2016;11(5):e0155423 doi: 10.1371/journal.pone.0155423 27192154 10.1371/journal.pone.0155423 PMC4871557

[20] Siegman-Igra Y. The significance of urine culture with mixed flora. Curr Opin Nephrol Hypertens. 1994;3(6):656–9. . 7881993 10.1097/00041552-199411000-00017

[21] Walter FG, Gibly RL, Knopp RK, Roe DJ. Squamous cells as predictors of bacterial contamination in urine samples. Ann Emerg Med. 1998;31(4):455–8. . 9546013 10.1016/s0196-0644(98)70253-7

[22] Mohr NM, Harland KK, Crabb V, Mutnick R, Baumgartner D, Spinosi S, et al Urinary Squamous Epithelial Cells Do Not Accurately Predict Urine Culture Contamination, but May Predict Urinalysis Performance in Predicting Bacteriuria. Acad Emerg Med. 2016;23(3):323–30. doi: 10.1111/acem.12894 . 26782662 10.1111/acem.12894

[23] Geerts N, Jansz AR, Boonen KJ, Wijn RP, Koldewijn EL, Boer AK, et al Urine flow cytometry can rule out urinary tract infection, but cannot identify bacterial morphologies correctly. Clin Chim Acta. 2015;448:86–90. Epub 2015/07/01. doi: 10.1016/j.cca.2015.06.020 26123581 10.1016/j.cca.2015.06.020

[24] Yang CC, Yang SS, Hung HC, Chiang IN, Peng CH, Chang SJ. Rapid differentiation of cocci/mixed bacteria from rods in voided urine culture of women with uncomplicated urinary tract infections. J Clin Lab Anal. 2017;31(5). doi: 10.1002/jcla.22071 . 27859671 10.1002/jcla.22071 PMC6817067

[25] Frazee BW, Enriquez K, Ng V, Alter H. Abnormal urinalysis results are common, regardless of specimen collection technique, in women without urinary tract infections. J Emerg Med. 2015;48(6):706–11. Epub 2015/04/07. doi: 10.1016/j.jemermed.2015.02.020 25841289 10.1016/j.jemermed.2015.02.020

[26] Brilha S, Proenca H, Cristino JM, Hanscheid T. Use of flow cytometry (Sysmex) UF-100) to screen for positive urine cultures: in search for the ideal cut-off. Clin Chem Lab Med. 2010;48(2):289–92. Epub 2009/12/08. doi: 10.1515/CCLM.2010.047 19961394 10.1515/CCLM.2010.047

[27] Evans R, Davidson MM, Sim LR, Hay AJ. Testing by Sysmex UF-100 flow cytometer and with bacterial culture in a diagnostic laboratory: a comparison. J Clin Pathol. 2006;59(6):661–2. Epub 2006/05/30. doi: 10.1136/jcp.2005.032847 PubMed Central PMCID: PMC1860387. 16731608 10.1136/jcp.2005.032847 PMC1860387

[28] Geerts N, Boonen KJ, Boer AK, Scharnhorst V. Cut-off values to rule out urinary tract infection should be gender-specific. Clin Chim Acta. 2016;452:173–6. Epub 2015/12/01. doi: 10.1016/j.cca.2015.11.022 26616731 10.1016/j.cca.2015.11.022

[29] Monsen T, Ryden P. A new concept and a comprehensive evaluation of SYSMEX UF-1000i flow cytometer to identify culture-negative urine specimens in patients with UTI. Eur J Clin Microbiol Infect Dis. 2017 doi: 10.1007/s10096-017-2964-1 ; PubMed Central PMCID: PMC5554267. 28386705 10.1007/s10096-017-2964-1 PMC5554267

[30] Moshaver B, de Boer F, van Egmond-Kreileman H, Kramer E, Stegeman C, Groeneveld P. Fast and accurate prediction of positive and negative urine cultures by flow cytometry. BMC Infect Dis. 2016;16(1):211 Epub 2016/05/18. doi: 10.1186/s12879-016-1557-4 PubMed Central PMCID: PMC4869392. 27189024 10.1186/s12879-016-1557-4 PMC4869392

[31] Jolkkonen S, Paattiniemi EL, Karpanoja P, Sarkkinen H. Screening of urine samples by flow cytometry reduces the need for culture. J Clin Microbiol. 2010;48(9):3117–21. Epub 2010/07/02. doi: 10.1128/JCM.00617-10 PubMed Central PMCID: PMC2937741. 20592157 10.1128/JCM.00617-10 PMC2937741

[32] de Boer FJ, Gieteling E, van Egmond-Kreileman H, Moshaver B, van der Leur SJ, Stegeman CA, et al Accurate and fast urinalysis in febrile patients by flow cytometry. Infect Dis (Lond). 2017;49(5):380–7. doi: 10.1080/23744235.2016.1274048 . 28077007 10.1080/23744235.2016.1274048

[33] Moja L, Kwag KH, Lytras T, Bertizzolo L, Brandt L, Pecoraro V, et al Effectiveness of computerized decision support systems linked to electronic health records: a systematic review and meta-analysis. Am J Public Health. 2014;104(12):e12–22. doi: 10.2105/AJPH.2014.302164 ; PubMed Central PMCID: PMC4232126. 25322302 10.2105/AJPH.2014.302164 PMC4232126

[34] Paattiniemi EL, Karumaa S, Viita AM, Karpanoja P, Makela M, Isojarvi J, et al Analysis of the costs for the laboratory of flow cytometry screening of urine samples before culture. Infect Dis (Lond). 2017;49(3):217–22. doi: 10.1080/23744235.2016.1239028 . 27766919 10.1080/23744235.2016.1239028

